# Radiolabeling of DOTA-like conjugated peptides with generator-produced ^68^Ga and using NaCl-based cationic elution method

**DOI:** 10.1038/nprot.2016.060

**Published:** 2016-05-12

**Authors:** Dirk Mueller, Wouter A P Breeman, Ingo Klette, Michael Gottschaldt, Andreas Odparlik, Manfred Baehre, Izabela Tworowska, Michael K Schultz

**Affiliations:** 1 University Hospital Halle (Saale), Department of Nuclear Medicine, Halle, Germany; 2 Department of Nuclear Medicine, Erasmus MC Rotterdam, Rotterdam, the Netherlands; 3 Zentralklinik Bad Berka, Department of Nuclear Medicine/PET Center, Bad Berka, Germany; 4 Friedrich Schiller University of Jena, Institute of Organic and Macromolecular Chemistry, Jena, Germany; 5 Jena Center for Soft Matter (JCSM), Jena, Germany; 6 RadioMedix, Inc., Houston, Texas, USA; 7 Departments of Radiology and Radiation Oncology (Free Radical Radiation Biology Program), University of Iowa, Iowa City, Iowa, USA

## Abstract

Gallium-68 (^68^Ga) is a generator-produced radionuclide with a short half-life (*t*_½_ = 68 min) that is particularly well suited for molecular imaging by positron emission tomography (PET). Methods have been developed to synthesize ^68^Ga-labeled imaging agents possessing certain drawbacks, such as longer synthesis time because of a required final purification step, the use of organic solvents or concentrated hydrochloric acid (HCl). In our manuscript, we provide a detailed protocol for the use of an advantageous sodium chloride (NaCl)-based method for radiolabeling of chelator-modified peptides for molecular imaging. By working in a lead-shielded hot-cell system, ^68^Ga^3+^ of the generator eluate is trapped on a cation exchanger cartridge (100 mg, ∼8 mm long and 5 mm diameter) and then eluted with acidified 5 M NaCl solution directly into a sodium acetate-buffered solution containing a DOTA (1,4,7,10-tetraazacyclododecane-1,4,7,10-tetraacetic acid) or DOTA-like chelator-modified peptide. The main advantages of this procedure are the high efficiency and the absence of organic solvents. It can be applied to a variety of peptides, which are stable in 1 M NaCl solution at a pH value of 3–4 during reaction. After labeling, neutralization, sterile filtration and quality control (instant thin-layer chromatography (iTLC), HPLC and pH), the radiopharmaceutical can be directly administered to patients, without determination of organic solvents, which reduces the overall synthesis-to-release time. This procedure has been adapted easily to automated synthesis modules, which leads to a rapid preparation of ^68^Ga radiopharmaceuticals (12–16 min).

## Introduction

^68^Ga-labeled compounds for molecular PET imaging are of increasing interest in nuclear medicine. DOTA or NOTA (1,4,7-triazacyclononane-1,4,7-triacetic acid)-conjugated peptides have a particularly important role as precursor molecules for the labeling with ^68^Ga. The well-known ^68^Ga-labeled somatostatin analogs DOTATOC (DOTA-[Tyr^3^]-octreotide) and DOTATATE (DOTA-[Tyr^3^]-octreotate) are particularly well suited for PET imaging of neuroendocrine tumors expressing somatostatin receptors^1^.

The predominant advantage of ^68^Ga radiopharmaceuticals is that the synthesis is based on generator-produced ^68^Ga and that it can be performed on site (and on demand), without the need for a medical cyclotron. Nowadays, ^68^Ge/^68^Ga generator systems are widely available, and they consistently deliver high-purity ^68^Ga for radiolabeling reactions. The parent radionuclide ^68^Ge is accelerator-produced through the ^69^Ga(p,2n) reaction, whereby Ga_2_O_3_ is used as the target material^2^.

Several ^68^Ge/^68^Ga generators have been developed in which ^68^Ge is adsorbed as a tetravalent compound onto an inorganic or organic matrix. The parent radionuclide decays with a half-life of 271 d to the trivalent ^68^Ga^3+^ cation. Because of distinct chemical differences, the daughter nuclide ^68^Ga^3+^ can be easily separated by elution from the generator with aqueous HCl. Depending on the carrier material (generator stationary phase), the concentration of eluent HCl varies from 0.05 to 1.0 M. The concentration of the parent nuclide ^68^Ge in the eluent in currently available generators has been found to be acceptably low, and it has been thoroughly investigated^3^. These generators deliver ^68^Ga in a high chemical purity with a low concentration of foreign ions. Heavy-metal ions such as iron and copper ions have a high impact on the labeling efficiency, as does zinc. Most important are iron impurities in the generator eluate or in the labeling reagents, which therefore must be avoided.

Numerous methods have been developed to synthesize ^68^Ga-labeled peptides starting from ^68^Ga elution from the generators. These methods can be classified into six different categories (Table 1). All use the amphoteric behavior of ^68^Ga to form cationic and anionic species such as cationic ^68^Ga^3+^ and anionic tetrachlorogallate [^68^GaCl_4_]^−^. Here we briefly describe the approach of each of these five classes of ^68^Ga radiolabeling paradigms and we provide a detailed protocol for the use of a NaCl-based method (class 6) for radiolabeling of chelator-modified peptides and small molecules for molecular imaging applications.

The first method known from literature is based on enrichment of ^68^Ga using a (tin(IV)-oxide) SnO_2_-based generator. The fraction of the eluate with the highest concentration of activity (e.g., in 1–2 ml) is buffered for labeling, and a final purification step using a solid-phase extraction (SPE; e.g., C18 cartridge) is required to trap the radiopeptide^4^. This purification step removes buffer compounds such as HEPES and unreacted ‘free’ ^68^Ga, which are unwanted in the final product, and it also reduces the ^68^Ge concentration resulting from small quantities of ^68^Ge breakthrough from the generator. The final (highly pure) product is elutable from the SPE cartridge, usually with a small volume of ethanol, and the mixture can be diluted with 0.9% (wt/wt) NaCl solution.

In a second approach, the labeling begins by eluting the generator and converting the eluted ^68^Ga^3+^ into the anionic [^68^GaCl_4_]^−^ species by the addition of concentrated HCl (final HCl concentration 5.5 M) and trapping anionic ^68^Ga on an anion exchanger^5^. The excess of HCl is removed from the anion exchanger cartridge using a stream of inert gas. [^68^GaCl_4_]^−^ can then be eluted with a small volume of water (0.2–0.4 ml) into the buffered precursor-containing reaction vial. Basically, the pH for the labeling of DOTA-conjugated, NOTA-conjugated or similarly conjugated peptides should be adjusted to between 3 and 4 (ref. 4). The labeling can be performed at 85–95 °C for DOTA-bearing peptides or at room temperature (21 °C) for, e.g., NOTA-conjugated compounds^6^. The use of concentrated HCl may be a disadvantage in the radiopharmaceutical practice.

A third approach to obtain ^68^Ga-labeled tracers uses a cation exchanger to trap cationic ^68^Ga^3+^ directly from the generator eluate. The adsorbed ^68^Ga can be eluted subsequently with mixtures of HCl-containing organic solvents (e.g., acetone/HCl) into the precursor peptide solution^7,8^. The organic solvent must be removed by heating the reaction mixture at 95 °C, and the labeled compound is separated using SPE cartridges. The resulting final product acetone concentrations are found to be well within regulatory limits for release. Labeling procedures using ethanol instead of acetone have also been published^9,10^. Because of the high concentration of ethanol in the radiopharmaceutical product, the determination of ethanol content is required in many countries. This method is patented and licensed for Eckert & Ziegler.

The fifth method for the utilization of ^68^Ga combines the high efficiency of the cationic concentration with the anionic purification. In this method, concentrated HCl is substituted by a small volume of diluted HCl. Organic solvents are not required^11,12^. This procedure delivers ^68^Ga in a high chemical and radiochemical purity, which allows the labeling of peptides at high specific activity. However, the requirement for two cartridges has limited the routine use of this method in practice.

All these methods are limited in their use for the routine production of radiopharmaceuticals, either because of the use of organic solvents or because of the use of semiconcentrated, corrosive-acting HCl.

**Figure 1:**
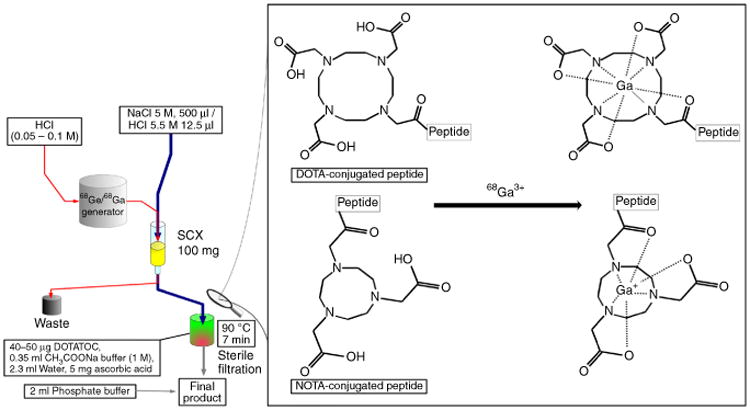
Schematic drawing of the NaCl-based labeling procedure for the labeling of DOTA- or NOTA-conjugated peptides with ^68^Ga.

In contrast, the procedure described in this protocol focuses on a ^68^Ga radiolabeling procedure (method 6 in Table 1), which requires the use of only one single cation exchanger cartridge, and purification of ^68^Ga for radiolabeling reactions is achieved just by manipulating cationic/anionic speciation of ^68^Ga. The method, pioneered by Mueller *et al*., uses the behavior of ^68^Ga to form anionic species present in concentrated NaCl solution^13^. In this method, ^68^Ga^3+^ of the generator eluate (usually in 0.1 M HCl or a lower concentration of HCl) is trapped on a cation exchange resin. Rinsing the column with acidified 5 M NaCl solution transforms the cationic ^68^Ga^3+^instantly and *in situ*, which results in desorption and elution of ^68^Ga as anionic [^68^GaCl_4_]^−^. This eluate is directed to a buffered solution that contains a chelator-modified DOTA-like peptide for labeling (see Fig. 1). The present protocol has been optimized for the use of DOTA- and NOTA-conjugated peptides^12–16^. The main advantages of this procedure are the high efficiency of desorption of the column and the absence of organic solvents.

Usually, the NaCl eluate of the cation-exchange resin is added to the reaction mixture, which contains 2–3 ml of water, and the NaCl concentration is therefore ∼1 M. Some proteins might be sensitive to high NaCl concentrations, and these usually precipitate by dehydration^17^. For many proteins and peptides, however, NaCl concentrations up to 1 M show stabilizing effects^18–20^. We recommend that preliminary experiments (i.e., appropriate biochemical assays) be performed to check that radiolabeling does not alter the biological activity of the peptides or proteins; if this labeling procedure is used to label compounds that are known to be sensitive, the binding affinity of the labeled products should be checked before routine production.

After labeling, neutralization and sterile filtration of the reaction mixture, quality control can be performed, and the ^68^Ga-labeled radiopharmaceutical can be directly administered to patients. Appropriate quality control for this process is iTLC, HPLC and measurement of pH value. It is not necessary to determine the concentration of organic solvents, which reduces the overall synthesis-to-release time and simplifies the overall process for radiolabeling and quality control for ^68^Ga-labeled radiopharmaceuticals. Furthermore, this procedure has been adapted easily to an automated synthesis module (Supplementary Fig. 1), thus leading to a rapid preparation of ^68^Ga-labeled radiopharmaceuticals (14 min; ref. 14).

## Materials

### Reagents

▲ **CRITICAL** Selection of ultra-high-purity reagents is crucial to the success of ^68^Ga labeling procedures. Practitioners should carefully select reagents with the lowest possible metal content (e.g., trace metal grade) to avoid unwanted interference in the ^68^Ga-DOTA labeling reaction. Examples of precise catalog numbers for trace metal grade reagents are given in this list of reagents. Reagents that are accompanied with a certificate of analysis stating metal concentrations at the parts per trillion level are preferred.

- Water for injection (e.g., Fresenius Kabi, cat. no. B101216)
- Hydrochloric acid (HCl) solution, >37% (e.g., TraceSELECT; Fluka, cat. no. 84415)
- Sodium chloride (NaCl), 99.99% (e.g., Suprapur; EMD Millipore/Merck Millipore, cat. no. 106406)
- Water, ultrapure (e.g., EMD Millipore/Merck Millipore, cat. no. 101262)
- Sodium acetate, ≥99.999% (e.g., TraceSELECT; FLUKA, cat. no. 59929)
- Acetic acid, 100% (e.g., EMD Millipore/Merck Millipore, cat. no. 100063)
- Sodium di-hydrogen phosphate dihydrate for analysis (e.g., EMSURE Reag. Ph Eur; EMD Millipore/Merck Millipore, cat. no. 106342)
- Di-sodium hydrogen phosphate dodecahydrate for analysis (e.g., EMSURE Reag. Ph Eur; EMD Millipore/Merck Millipore, cat. no. 106579)
- l-Ascorbic acid ACS reagent, ≥99% (e.g., Sigma-Aldrich, cat. no. 255564)
- Trifluoroacetic acid for spectroscopy (e.g., Uvasol (TFA); EMD Millipore/Merck Millipore, cat. no. 108262)
- Acetonitrile for preparative chromatography (e.g., Prepsolv; EMD Millipore/Merck Millipore, cat. no. 113358)
- Ammonium acetate for analysis (e.g., EMSURE Reag. Ph Eur; EMD Millipore/Merck Millipore, cat. no. 101116)
- Methanol for analysis (e.g., EMSURE Reag. Ph Eur; EMD Millipore/Merck Millipore, cat. no. 106009)
- Colorimetric iron test (e.g., Merck Millipore, cat. no. 114759)
- ▲ **CRITICAL** Use this test in parallel to check your reagents and equipment for the presence of iron contamination (Box 1).
- Colorimetric copper test (e.g., Merck Millipore, cat. no. 114765)
  ▲ **CRITICAL** Use this test in parallel to check your reagents and equipment for the presence of copper contamination.
- Colorimetric zinc test (e.g., Merck Millipore, cat. no. 114780)
  ▲ **CRITICAL** Use this test in parallel to check the eluate of the generator for the presence of zincions after the complete decay of ^68^Ga.
- iTLC eluent (Box 2)
- HPLC solvent A (Box 2)
- HPLC solvent B (Box 2)
- DOTA-like conjugated peptide (e.g., piChem, Bachem); DOTA-TOC, DOTA-NOC, DOTA-TATE, NODAGA-THERANOST and DOTA-SB3 are commonly used. Peptides carrying chelators that are different from DOTA or NOTA should be carefully checked for labeling efficiency when applying this protocol (e.g., for DATA-TOC, it was reported that this protocol led to incomplete labeling^10^).

### Equipment

▲ **CRITICAL** The equipment and apparatus presented here are for informational purposes. For example, several manufacturers offer variants of the dose calibrator presented in this list that would be suitable for measurement of the final product radioactivity concentration. The cation-exchange and reverse-phase cartridges that are presented were available at the time of writing and were effective in our method development and evaluations presented here.

- Bond Elut SCX Cartridges, 100 mg, particle size 40 μm (Agilent, cat. no. 12102013)
- Accument basic pH meter (Fisher, cat. no. 13-636-AB15PB)
- CRC-25PET Dose Calibrator (Capintec, cat. no. 5130-3215)
- Sep-Pak C18 Plus Short Cartridge, 360 mg sorbent per cartridge, 55–105 μm particle size (Waters, cat. no. WAT020515; this cartridge is commonly not required, and it is only relevant if a final purification is needed; Table 1)
- Ethanol 96% (e.g., EMD Millipore/Merck Millipore, cat. no. 1.00971)
- Reaction vial (e.g., Wheaton V vial 10 ml, diameter 24 mm, cat. no. W015285), alternatively an evacuated vial 10 ml is also applicable (e.g., Mallinckrodt, Evacuated Vials for use with UltraTechnekow 10 ml, cat. no. N784A0)
- Heating block (e.g., Biostep, metal block thermostat, cat. no. GT53-S0001, metal block, *d* = 25 mm (reaction vial dependent), cat. no. F3509)
- Sterile syringe filter: Millex-GV Syringe Filter Unit, 0.22 μm, PVDF, 33 mm (EMD Millipore/Merck Millipore, cat. no. SLGV033RS), or sterile syringe filter, ultra-low protein binding, Cathivex-GV 25 mm, 0.22 μm, PVDF (EMD Millipore/Merck Millipore, cat. no. SLGV0250S)
- Luer-Lok connector for the SCX cartridge (neoLab (http://www.myneolab.de), Connector, LL female/olive 4.0–5.0 mm, cat. no. 21889)

### Reagent Setup

**0.1 M HCl** 0.1 M HCl is used for the elution of the ^68^Ge/^68^Ga generator (Eckert & Ziegler). Dilute 8.3 ml of 37% HCl with water (Ultrapure) to 1,000 ml. Can be stored at room temperature (21 °C); stable for 1 year.

**0.05 M HCl** 0.05 M HCl is used for the elution of the ^68^Ge/^68^Ga generator (ITM Isotope Technologies Munich AG). Dilute 4.2 ml of 37% HCl with water (Ultrapure) to 1,000 ml. Can be stored at room temperature (21 °C); stable for 1 year.

**0.6 M HCl** 0.6 M HCl is used for the elution of the ^68^Ge/^68^Ga generator (iThemba ^68^Ge/^68^Ga generator, IDB Holland bv). Dilute 50 ml of 37% HCl with water (Ultrapure) to 1,000 ml. Can be stored at room temperature (21 °C); stable for 1 year.

**5.5 M HCl solution** 5.5 M HCl solution is used for the preconditioning of the SCX cartridge. Mix 23 ml of 37% HCl in 27 ml of water (Ultrapure). Can be stored at room temperature (21 °C); stable for 1 year.

**5 M NaCl/HCl solution** 5 M NaCl/HCl solution is used for the elution of ^68^Ga from the SCX cartridge. Mix 7.3 g of NaCl and 625 μl of 5.5 M HCl in 25 ml of water (Ultrapure). Can be stored at room temperature (21 °C); stable for 1 year.

**1.4% ascorbic acid solution** 1.4% ascorbic acid solution is used to reduce radiolysis in this protocol. The ascorbic acid solution should be freshly prepared.

**1 M sodium acetate buffer, pH 4.5** 1 M sodium acetate buffer, pH 4.5, is used for the labeling of DOTA-like conjugated peptides in this protocol. Mix 4.1 g of sodium acetate in 50 ml of water (Ultrapure) and 1 ml of concentrated HCl (37%), adjusted with acetic acid (100%) to a pH of 4.5. Can be stored at room temperature (21 °C); stable for 1 year.

**Reaction solution** Reaction solution is used for the labeling of the DOTA-like conjugated peptide. Mix 25–35 nmol of the DOTA-like conjugated peptide (e.g., 40–50 μg of the DOTATOC or DOTATATE) and 0.35 ml of 1 M sodium acetate buffer (pH = 4.5) in water (e.g., 0.3–2.5 ml). To reduce radiolysis of the radiolabeled peptide, the reaction mixture should also contain a radical scavenger such as 0.35 ml of 1.4% ascorbic acid solution. The reaction solution should be freshly prepared.

**Phosphate buffer** Phosphate buffer for the final neutralization is used in this protocol. Mix 3.05 g of disodium monohydrogen phosphate dodecahydrate (Ph. Eur.) and 0.462 g of sodium dihydrogen phosphate dihydrate (Ph. Eur.) in 20 ml of water for injection. Can be stored at room temperature (21 °C); stable for 1 year.

**Figure 2:**
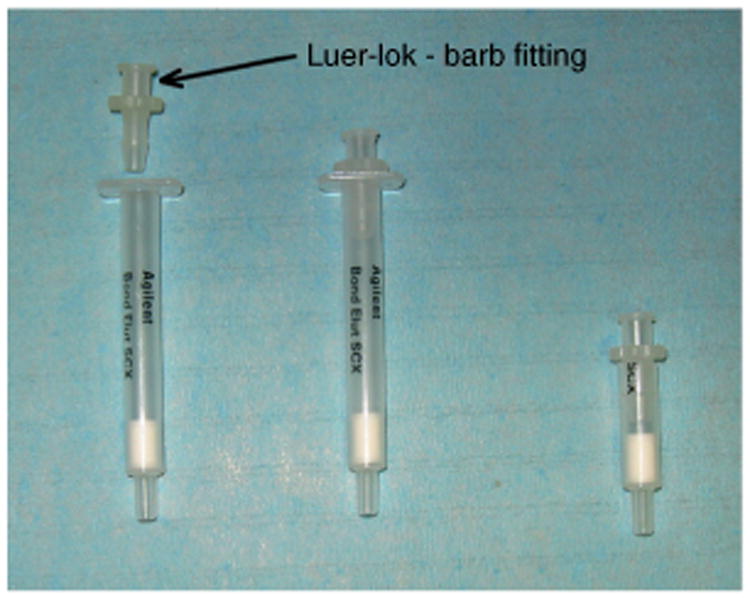
Preconditioning of the SCX cartridge, SCX cartridge with Luer-lock barb fitting.

**Preconditioning of the SCX cartridge** Preconditioning of the SCX cartridge used for trapping of ^68^Ga of the ^68^Ge/^68^Ga generator eluate: if necessary, cut the SCX cartridge. Plug in the Luer-lok barb fitting to the SCX cartridge or the cutoff cartridge, as shown in Figure 2. Rinse the SCX cartridge with 1 ml of 5.5 M HCl and subsequently with 10 ml of water for injection.

### Equipment Setup

**Generators** This procedure will work with a variety of different generators. We describe the specifications and elution conditions for three generators that we have experience with. **! CAUTION** ^68^Ga emits both positrons and gamma rays. It is imperative that employees using these procedures observe the guidelines set forth by their institution and the Nuclear Regulatory Commission, and that they observe ALARA (as low as reasonably achievable) protocols to minimize radiation exposure. When handling any radioactive material, proper protective equipment, shielding, body and ring dosimetry badges, and a survey meter are required.

**Eckert & Ziegler** This ^68^Ge/^68^Ga generator can be ordered from Eckert & Ziegler Radiopharma Segment Headquarters (for sales offices world-wide please contact info.eurotope@ezag.de). As dictated by the manufacturer, this generator is eluted with 6 ml of 0.1 M HCl. The generator eluate can be transferred directly through the SCX cartridge. **! CAUTION** ^68^Ga emits both positrons and gamma rays; refer to the precautionary advice above.

**ITM Isotope Technologies Munich AG** This ^68^Ge/^68^Ga generator (itG ^68^Ge/^68^Ga generator) can be ordered from ITM Isotope Technologies Munich AG (for sales offices world-wide please contact sales@itg-garching.de). As dictated by the manufacturer, the generator is eluted with 0.05 M HCl. The generator eluate can be transferred directly through the SCX cartridge. **! CAUTION** ^68^Ga emits both positrons and gamma rays; refer to the precautionary advice above.

**IDB Holland bv** This ^68^Ge/^68^Ga generator (iThemba ^68^Ge/^68^Ga generator) can be ordered from IDB Holland bv (for sales offices world-wide please visit http://www.idb-holland.com/gallium-68-generator/, www.sales@idb-holland.com). As dictated by the manufacturer, the generator is eluted with 0.6 M HCl. **! CAUTION** ^68^Ga emits both positrons and gamma rays; refer to the precautionary advice above.

▲ **CRITICAL** The generator eluate must be diluted with five times the volume with water before transferring the eluate through the SCX cartridge.

## Procedure

### Labeling and quality control of DOTA-like peptides with ^68^Ga ● TIMING 12–16 min

**! CAUTION** The adsorbed ^68^Ga emits both positrons and gamma rays. It is imperative that employees using these procedures observe the guidelines set forth by their institution and the Nuclear Regulatory Commission, and that they observe ALARA protocols to minimize radiation exposure. When you are handling any radioactive material, proper protective equipment, shielding, body and ring dosimetry badges, and a survey meter should be used appropriately according to these guidelines.

▲ **CRITICAL** Check all the reagents, tips, caps and reaction vessels and the ^68^Ge/^68^Ga generator eluate in parallel for the presence of iron contamination (Box 1).

**1**| Elute the^68^Ge/^68^Ga generator with 6 ml of aqueous HCl. The concentration of HCl for eluting the generator depends on the type of generator (see ‘Generators’ in Equipment Setup section and Reagent Setup). A flow rate of ∼2 ml per min elutes ^68^Ga from the generator.

**2**| If the concentration of HCl used is 0.6 M (iThemba ^68^Ge/^68^Ga generator), dilute the ^68^Ge/^68^Ga generator eluate with 30 ml of water.

**3**| Transfer the eluate through the SCX cartridge. If the concentration of HCl for eluting the generator is 0.1 M or lower (depending on the used generator), the eluate of the generator can be transferred directly through the SCX cartridge by connecting the outlet of the ^68^Ge/^68^Ga generator with the SCX cartridge.

**4**| Detach the SCX cartridge and dry it with a stream of air.

**5**| Elute the ^68^Ga, which is trapped onto the SCX, with 0.5 ml of 5 M NaCl/HCl solution into the reaction vial, which contains the reaction solution; the final pH in the reaction vial should be pH 3–4. Incubate the reaction mixture at 85–95 °C for 8–12 min.

**6**| (Optional) Determine the optimal reaction time by analyzing 1- to 2-μl aliquots of the reaction mixture by radio-iTLC or radio-HPLC, as described in Box 2.

▲ **CRITICAL STEP** We recommend that this initial experiment be done so as to achieve the required radiochemical purity (% incorporated ^68^Ga) for final product release.

▲ **CRITICAL STEP** For radio-iTLC, a 1- to 2-μl aliquot of the reaction mixture can be directly applied to the iTLC plate.

**7**| Determine the efficiency of the reaction. To do this, remove a 1- to 2-μl aliquot of the reaction mixture and analyze it by radio-iTLC or radio-HPLC, as described in Box 2.

**? TROUBLESHOOTING**

**8**| At the end of the reaction period—after you are satisfied that the reaction has gone to completion or you have completed the steps in the Troubleshooting table—add 2 ml of sterile sodium phosphate buffer to the reaction mixture to adjust the pH of the solution to 6–7.

**? TROUBLESHOOTING**

**9**| Pass the reaction mixture through a sterile filter, as mentioned in the equipment list.

If the reaction mixture reaches a radiochemical purity (expressed as % of incorporation of activity in the DOTA-like peptide) that meets the stated release criterion (typically >95%) and the solution has been sterile filtered, the final product may be diluted further for dose administration to any desired volume for animal or patient studies, respectively.

**■ PAUSE POINT**

**? TROUBLESHOOTING**

**10**| The stability of the final product at room temperature should be investigated in a separate labeling experiment by radio-HPLC. If a radical scavenger is used, the labeled peptide can be usually applied within 4 h. For a clinical routine production of ^68^Ga-labeled radiopharmaceuticals, it has been shown that a time distance of a minimum of 2 h after the first elution is sufficient for starting a second radiolabeling reaction. As described in the ANTICIPATED RESULTS, the decay of ^68^Ge of the generator delivers enough new ^68^Ga for the second synthesis.

**? TROUBLESHOOTING**

Troubleshooting advice can be found in table 2.

## ● Timing

Steps 1–10, labeling and quality control of DOTA-like peptides with ^68^Ga: 12–16 min

Box 1, determination of iron: ∼6 min

Box 2, measurement of radiolabeling efficiency: iTLC (option A), ∼6 min; HPLC (option B), 10–15 min

## Anticipated Results

**Figure 3:**
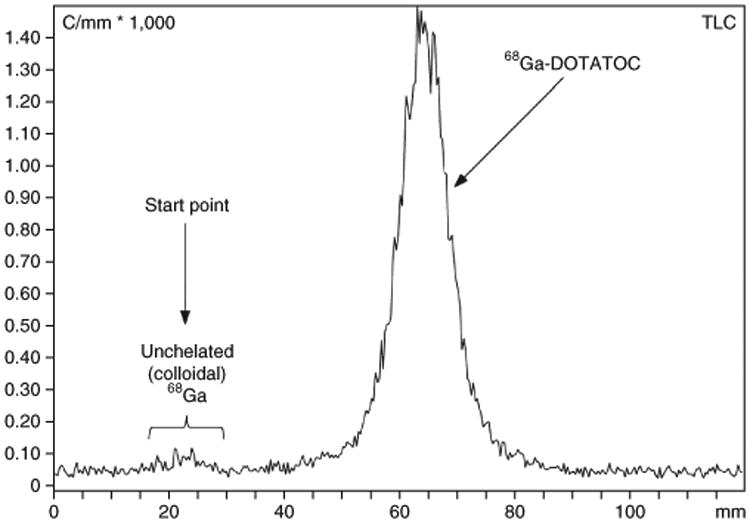
An example of a radio-iTLC chromatogram of [^68^Ga]-DOTATOC using the iTLC method described in Box 2 (mobile phase consisting of 1 M ammonium acetate/methanol 1:1 vol/vol). The ^68^Ga-labeled peptide has an approximate retardation factor of 0.8–1.0.

Our method for the labeling of DOTA-like conjugated peptides with ^68^Ga has been published in earlier publications^13,14^. This labeling procedure was routinely used in >1,000 synthesis runs, for >3,000 patient scans and in several nuclear medicine facilities. If the labeling is carried out in the absence of heavy-metal contaminations (especially iron and copper), the probability of success using this protocol is high. Typically, the labeling efficiency of the ^68^Ga-labeled DOTA-like conjugated peptides should be >95% within 12 min at 90 °C. By using the above-mentioned iTLC method, *R*_f_ values of ∼0.8–1.0 are obtained for the labeled peptide, whereas any unchelated ^68^Ga (colloidal) remains at the start point of the TLC plate or migrates with a retardation factor of ∼*R*_f_ = 0–0.1 (Fig. 3). In the case of a radiopharmaceutical production of a ^68^Ga-labeled peptide (e.g., for patient doses), the iTLC method may need to be changed to adhere to current local pharmaceutical regulations.

**Figure 4:**
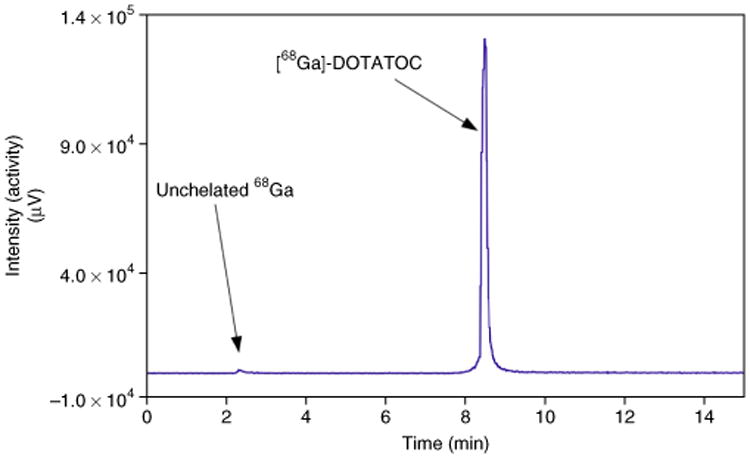
An example of a HPLC chromatogram of [^68^Ga]-DOTATOC using the HPLC system previously described, a mobile phase consisting of 0.1% TFA in 5% acetonitrile/water (solution A) and 0.01% TFA in 95% acetonitrile/water (solution B) and a gradient from 0–2 min 100% A, 2–15 min to 100% B (flow rate: 1.2 ml/min). The ^68^Ga-labeled peptide has an approximate retention time of 9 ± 3 min (refs. 13,14).

Depending on the hydrophobicity or hydrophilicity of the peptide, the HPLC column and the gradient applied, the retention times for different ^68^Ga-labeled peptides will vary. A typical HPLC chromatogram of the ^68^Ga-labeled peptide ^68^Ga-DOTATOC is shown in Figure 4. By using the above-mentioned HPLC system and HPLC method, the ^68^Ga-labeled peptide has an approximate retention time of 9 ± 3 min, and it can be determined by the radiodetector of the HPLC. Any nonchelated ^68^Ga should be detected in the first 1–3 min of the run at a flow rate of 1.2 ml/min (void volume).

### Elution behavior of the ^68^Ge/^68^Ga generator

**Figure 5:**
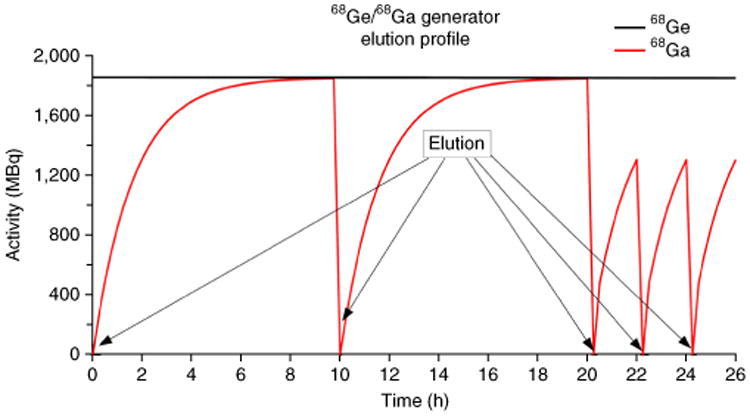
Elution profile of the ^68^Ge/^68^Ga generator and formation of ^68^Ga. 10 h after the former elution, there is an equilibrium of formation of ^68^Ga and decay to ^68^Zn, and the maximum of the elutable activity of ^68^Ga is reached. By two half-lives after the former elution, 75% of the maximum ^68^Ga activity is already elutable from the generator.

After elution of the ^68^Ge/^68^Ga generator, new ^68^Ga will be formed by decay of the parent radionuclide ^68^Ge. The growth of ^68^Ga subsequent to elution follows first-order kinetics described by well-known radioactive daughter growth equations. Thus, elution of ^68^Ga after 68 min results in an approximate activity that is 50% of the total ^68^Ge present in the column. It follows then that, after 136 min (two half-lifes of ^68^Ga), the expected elution of ^68^Ga is 75% of total ^68^Ge present, which is a useful activity of ^68^Ga to start a new synthesis run. After ∼10 h (10 half lives), >99% of the activity of ^68^Ga will have grown into equilibrium and may be eluted (Fig. 5).

**Figure 6:**
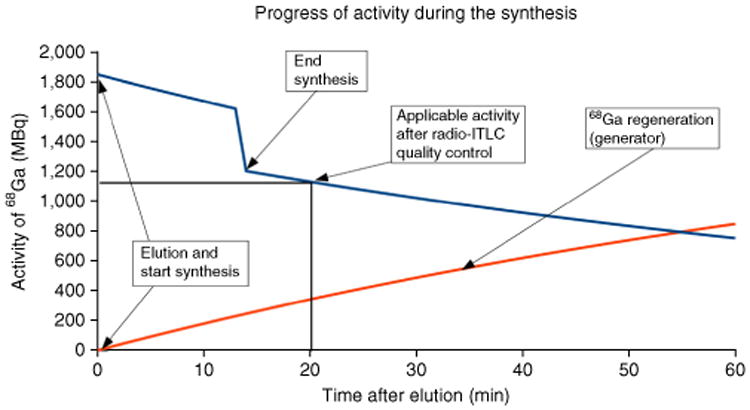
Progress of ^68^Ga activity during one automated synthesis run for the routine pharmaceutical production of ^68^Ga-DOTATOC—synthesis time: 14 min, time until application: 20 min (vertical black line), start activity: 1.85 GBq, activity after synthesis: 1.20 GBq, applicable activity for patients: 1.13 GBq (horizontal black line). Blue line: progress of eluted activity of ^68^Ga that is used for the automated synthesis run. Red line: regeneration of ^68^Ga activity in the ^68^Ge/^68^Ga generator after elution. The loss of activity at the end of the synthesis results from the loss of final product due to adsorption processes during the sterile filtration and in the synthesis module and also because of the taken samples for the quality control.

An example of the progress of ^68^Ga activity after elution of the generator and start of an automated pharmaceutical synthesis run for the labeling of, e.g., DOTATOC, is shown in Figure 6. The synthesis is completed after 14 min, and an additional 6 min are required for the quality control (radio-iTLC, pH). Overall, the final radiopharmaceutical product is ready to be delivered to the clinics within 20 min from the start of the synthesis (including time for elution of the generator).

**Figure 7:**
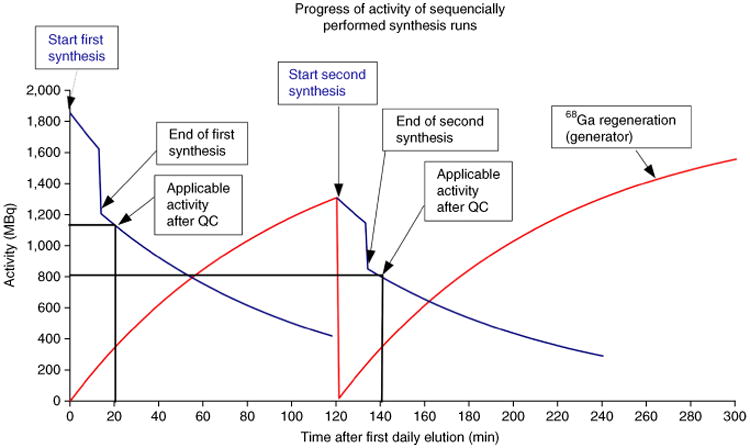
Progress of activity of sequentially performed synthesis runs for the routine pharmaceutical production of ^68^Ga-DOTATOC—first start activity: 1.85 GBq, activity after first synthesis: 1.20 GBq, first applicable activity (20 min, vertical black line): 1.13 GBq (horizontal black line), second start activity: 1.31 GBq, activity after second synthesis: 0.85 GBq, second applicable activity (140 min, vertical black line): 0.80 GBq (horizontal black line). Blue line: progress of eluted activity on ^68^Ga that is used for the automated synthesis run. Red line: regeneration of ^68^Ga activity in the ^68^Ge/^68^Ga generator after elution.

**Figure 8:**
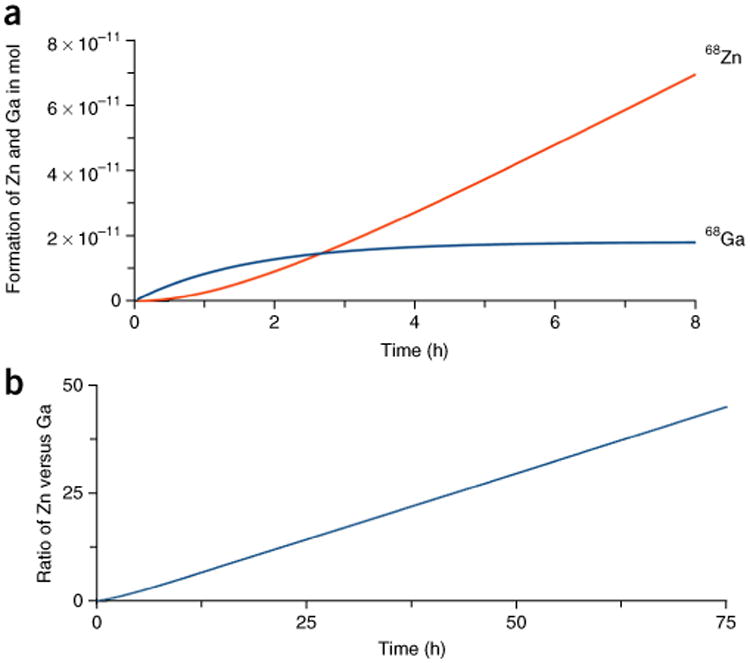
Decay of ^68^Ga and formation of ^68^Zn. (**a**) Calculated formation of ^68^Ga and ^68^Zn as *f* (time) in hours after elution of a 50 mCi (1.85 GBq) ^68^Ge/^68^Ga generator. The specific activity of ^68^Ga corresponds to 9.81 × 10^−15^ mol/MBq. (**b**) Calculated molar ratio of ^68^Zn versus ^68^Ga as *f* (time) in hours after post-prior elution of 50 mCi (1.85 GBq) ^68^Ga of a ^68^Ge/^68^Ga generator. In theory, 3 d after post-prior elution the molar ratio of [^68^Zn]/[^68^Ga] = 43.

For routine use of ^68^Ga-labeled peptides in clinical practice, a sequential production of ^68^Ga-labeled radiopharmaceuticals is often required. Figure 7 shows the expected activity in sequentially performed synthesis runs. While the second synthesis starts 2 h after the first elution, the start activity is ∼25% lower compared with the first daily synthesis run (Fig. 7). Importantly, radioactive decay of ^68^Ga produces measureable quantities of stable isotope ^68^Zn. The concentration of zinc in the generator eluate increases as a function of time after elution^4^, as shown in Figure 8a. High concentrations of zinc ions may interfere with ^68^Ga labeling reactions; therefore, the time between the last elution of the generator and the start of a radiolabeling experiment should not be longer than 48 h. Although the procedure that we describe here eliminates most cations (such as Zn), significant quantities of Zn indeed build continuously between generator elutions. Thus, it is prudent to minimize the potential for interference by frequent flushes of the generator to remove stable Zn and any other impurities that may be associated with the solid matrix generator materials. After 3 d post-prior elution of the ^68^Ga/^68^Ga generator, the calculated ratio of ^68^Zn versus ^68^Ga corresponds to 43 (Fig. 8b). The influence of zinc, iron, copper and other metal ions on labeling efficiency is described in detail in the literature^21,22^. The colorimetric test with thioacetic acid as standard procedure for the determination of critical concentrations of heavy metals, as described in the European Pharmacopoeia^23^, is a very useful tool for the routine quality control of the generator eluate. In some countries, the determination of the concentration of iron and zinc in the generator eluate is requested. The test kits, as mentioned in Reagents, are based on the formation of colored metal complexes with high extinction coefficients, and they allow the easy determination of iron and zinc in a range between 0.1 and 5 μg/ml.

## Figures and Tables

**Table 1:** Labeling methods.

	Method	Ion exchanger	Elution solution ion exchanger	Buffer	Advantages/limitations	References
1	Fractional elution	_	_	HEPES	(+) No preconcentration is required	4
					(−) Only ∼80% of activity is available for labeling	
					(−) Final purification is required	
					(−) The final product contains ethanol	
2	Anionic method	SAX	∼0.4 ml water	HEPES	(+) The method delivers ^68^Ga in high chemical purity	5
					(−) Handling with concentrated HCl is necessary	
					(−) Final purification is required	
					(−) The final product contains ethanol	
3	Cationic method (acetone)	SCX	0.5 ml of acetone/HCl	HEPES	(+) The method is well established	6
					(−) Final purification is required	
					(−) The final product contains ethanol and other side products	
					(±) The method is licensed for Eckert & Ziegler	
4	Cationic method (ethanol)	SCX	1 ml of ethanol/HCl	HEPES, ammonium acetate	(+) Because of the low concentration of foreign ions in the reaction mixture, high specific activities are achievable	8–10
					(−) The final product contains ethanol	
					(±) The method is licensed for Eckert & Ziegler	
5	Combined cationic-anionic method	(1) SCX(2) SAX	(1) 0.4 ml of 4 M HCl(2) ∼0.4 ml of water	HEPES	(+) Because of the low concentration of foreign ions in the reaction mixture, high specific activities are achievable	11,12
					(−) Handling of 7 M HCl is required	
					(−) The final product contains ethanol	
					(−) Two steps of postprocessing ^68^Ga eluate are needed	
6	Cationic method (NaCl)	SCX	0.5 ml of 5 M NaCl/0.1 M HCl	Ammonium acetate, sodium acetate	(+) No organic solvents are found in the final product	13,14
					(+) High specific activities are routinely achievable	
					(±) The reaction mixture usually contains 1 M sodium chloride, which could be disadvantageous for NaCl-sensitive peptides	

**Table 2:** Troubleshooting table.

step(s)	problem	solution
Steps 7 and 8	Unchelated ^68^Ga is discovered in the solution	Take a sample and measure its pH. The final pH during the reaction should be between 3 and 4. If the pH is <3, add sodium acetate buffer (Reagent Setup) to the reaction mixture stepwise in 100-ml aliquots until the pH is between 3 and 4. If the pH is higher than 4, adjust the pH with diluted HCl or use a lower amount of sodium acetate buffer for the next labeling so that the pH of the reaction mixture is between 3 and 4 After re-adjusting the pH of the reaction mixture, incubate the reaction solution for an additional 7 min and analyze an aliquot by radio-iTLC and/or radio-HPLC.
	Unchelated ^68^Ga is still present in the solution after control of the pH of the reaction mixture	Incubate the reaction solution for an additional 5 min and analyze another aliquot by radio-iTLC and/or radio-HPLC
	Unchelated ^68^Ga is still present in the solution after the additional incubation	Add 5–7 nmol DOTA-like conjugated peptide (if specific activity is not a main focus) and incubate for an additional 5 min. Analyze another aliquot by radio-iTLC and/or radio-HPLC
Steps 7–9	Unchelated ^68^Ga is still present in the solution after incubation with additional peptide	Apply the reaction mixture to an activated SPE (e.g., Sep-Pak) cartridge^a^. Wash the cartridge with 5 ml of water (for injection) and elute dropwise the ^68^Ga-labeled peptide with 0.5 ml of a 1:1 ethanol:saline solution
Steps 7 and 8	Unchelated ^68^Ga is present in the solution and you suspect that this is due to iron contamination of the reaction mixture	Check all used reagents for iron contamination (including the generator eluate and other components (e.g., reaction vial)) using a colorimetric test (Box 1) or an atomic absorption spectrometer. Replace the contaminated reagent or component. If iron is detectable on the preconditioned SCX cartridge, rinse the SCX cartridges before the labeling with 1 ml of HCl (5.5 M), wait 2 min, and rinse again with 1 ml of HCl (5.5 M) and then with 10 ml of water. If iron is detectable in the reaction vial, rinse the vial before the labeling with 1 ml of HCl (5.5 M) and then with 10–20 ml of water
Steps 7–10	Multiple radioactive peaks are observed from a sample that is known to be analytically pure and free of isomers	Radiolysis may be occurring. Add ∼5 mg of a radical scavenger to the original reaction buffer and start the labeling procedure again. Possible radical scavengers include ascorbic acid, ethanol, gentisic acid and methionine

a Sep-Pak purification of the labeled peptide: If a Sep-Pak C-18 cartridge is required to remove unreacted Ga^3+^, it should be activated by preconditioning with ethanol. For this purpose, rinse the cartridge with 5 ml of ethanol and then with 5 ml of water (‘for injection’ quality). Transfer the reaction mixture through the C-18 cartridge and rinse the cartridge again with 2 ml of water (‘for injection’ quality). After elution of the ^68^Ga-labeled peptide with ethanol, it is essential to either remove the organic solvent from the resulting solution or to dilute the solution with isotonic saline solution before using the labeled compound as a radiopharmaceutical for animal studies or patient administration. The solvent can be evaporated by allowing a gentle stream of inert gas (helium, argon or nitrogen) to pass over the solution. To avoid adherence of the labeled peptide onto the inner surface of the reaction vial, the solution should not be concentrated to dryness.

## Box 1 Determination of iron ● TIMING ∼6 min

Radiolabeling of tracers with ^68^Ga requires that the reagents, tips, caps and reaction vessels used be as free as possible of trace metals, especially iron. If required, metal-free plastic needles must be used during the labeling reaction (Non-Vented Spike, e.g., Qosina, cat. no. 11757 or 11656). The determination of iron contaminations in reagents, generator eluate or other components using an atom absorption spectrometer is possible, but the use of the colorimetric test as mentioned above in Reagents is very helpful and easy to handle. The colorimetric test allows determination of iron concentrations between 0.1 and 5 μg/ml. The concentration of iron during the labeling should be <0.1 μg/ml.

## Box 2 Measurement of radiolabeling efficiency ● TIMING iTLC (option a), ∼6 min HPLC (option B), 10–15 min

The radiolabeling efficiency can be measured by iTLC (option A) or by HPLC (option B). The HPLC shows also potential side products that can be formed by radiolysis.

### Additional materials

iTLC:

iTLC chamber

iTLC Paper (iTLC-SG; Agilent, cat. no. SGI001)

miniGITA positron Radio iTLC scanner (e.g., Raytest)

Eluent for ^68^Ga-labeled DOTA-like conjugated peptides used in this protocol: 1 M ammonium acetate/methanol (1:1 vol/vol)

### HPLC

HPLC pump: Jasco PU-1580; quaternary gradient unit: Jasco LG-1580-04

HPLC radio detector: biostep IsoScan LC

RP-18 column (e.g., LiChrospher 100 RP-18 endcapped (5 μm) LiChroCART 250-4, EMD Millipore/Merck Millipore, cat. no. 150838)

HPLC eluents for ^68^Ga-labeled DOTA-like conjugated peptides used in this protocol: solvent A: 5% acetonitrile in water (0.1% TFA); and solvent B: 95% acetonitrile in water (0.01% TFA)

HPLC method:

Flow rate: 1.2 ml/min

Equilibration time:	2 min	A: 100%	B: 0%
Start method:	0–2 min	A: 100%	B: 0%
Gradient:	2–15 min	A: 100–0%	B: 0–100%
	15–17 min	A: 0%	B: 100%
Gradient:	17–19 min	A: 0–100%	B: 100–0%
	19–21 min	A: 100%	B: 0%

### Procedure

1. Measurement of radiolabeling efficiency by various techniques

- **Measurement of radiolabeling efficiency by itlc**
  - Pour the iTLC eluent (Reagent Setup) into the iTLC chamber until it is 0.5 cm deep and covers the chamber.
  - Place a 1- to 2-μl spot of the sample of the final reaction mixture onto the start point of the iTLC strip (at the first centimeter of the iTLC strip above the level of the iTLC eluent in the chamber).
  - Place the iTLC strip upright in the iTLC chamber gently. Cover the chamber and allow the solvent to run up the support material.
  - When the solvent is ∼1 cm from the top of the strip, remove it from the chamber and dry the strip.
  - Place the strip into a thin plastic sheet (e.g., Glad Clingwrap) and lay it onto the radio-iTLC scanner in the appropriate orientation for the scan.
  - Scan the radio-iTLC and calculate the amount of unchelated ^68^Ga (*R*_f_ = 0–0.1) and the ^68^Ga-labeled peptide (*R*_f_ = 0.8–1.0).
- **Measurement of radiolabeling efficiency by Hplc**
  Take a sample (10–20 μl) of the final reaction solution and inject it on the RP-HPLC using the HPLC method as mentioned in this box.

*Preliminary step*. Usually, the sample can be injected directly without dilution using the HPLC radio detector, as mentioned in the Equipment Setup. Because of the pH of the reaction mixture (3μ4), a neutralization of the HPLC sample is not required. Care should be taken to optimize the aliquot volume and radioactivity concentration of the radio-HPLC aliquot to ensure that the radioactivity concentration does not overwhelm the radioactivity detector at the outflow of the radio-HPLC system. Preliminary experiments should be conducted to develop an understanding of detection efficiency and upper threshold of the detector before the preparation of a dose that will be used for important biological experiments or clinical work.

## References

[1] Menda Y (2013). Repeatability of gallium-68 DOTATOC positron emission tomographic imaging in neuroendocrine Tumors. Pancreas.

[2] (2010). Production of Long Lived Parent Radionuclides for Generators, 68Ge, 92Sr, 90Sr and 188W. http://www-pub.iaea.org/MTCD/publications/PDF/Pub1436_web.pdf.

[3] Velikyan I (2014). Prospective of ^68^Ga-radiopharmaceutical development. Theranostics.

[4] de Blois E (2011). Characteristics of SnO_2_-based ^68^Ge/^68^Ga generator and aspects of radiolabelling DOTA-peptides. Appl Radiat Isot.

[5] Meyer GJ, Mäcke H, Schuhmacher J, Knapp WH, Hofmann M (2004). ^68^Ga labelled DOTA-derivatised peptide ligands. Eur J Nucl Med Mol Imaging.

[6] Eder M (2014). Novel preclinical and radiopharmaceutical aspects of [^68^Ga]Ga-PSMA-HBED-CC: A new PET tracer for imaging of prostate cancer. Pharmaceuticals.

[7] Zhernosekov KP (2007). Processing of generator produced ^68^Ga for medical application. J Nucl Med.

[8] Roesch F, Baum RP, Roesch F (2012). Post-processing via cation exchange cartridges: versatile options. Theranostics, Gallium-68 and Other Radionuclides Recent Results in Cancer Research.

[9] Eppard E, Wuttke M, Nicodemus PL, Rösch F (2014). Ethanol-based post-processing of generator-derived 68Ga toward kit-type preparation of 68Ga-radiopharmaceuticals. J Nucl Med.

[10] Seemann J, Eppard E, Waldron BP, Ross TL, Roesch F (2015). Cation exchange-based post-processing of 68Ga-eluate: a comparison of three solvent systems for labelling of DOTATOC, NO2AP(BP) and DATA(m.). Appl Radiat Isot.

[11] Müller D, Klette I, Baum RP (2011). The combined cationic-anionic purifcation of the ^68^Ge/^68^Ga generator eluate for the labeling of fragile peptides. World J Nucl Med.

[12] Loktionova NS (2011). Improved column-based radiochemical processing of the generator produced 68Ga. Appl Radiat Isot.

[13] Mueller D (2012). Simplified NaCl based ^68^Ga concentration and labeling procedure for rapid synthesis of ^68^Ga radiopharmaceuticals in high radiochemical purity. Bioconjug Chem.

[14] Schultz MK, Mueller D, Baum RP, Leonard Watkins G, Breeman WAP (2013). A new automated NaCl based robust method for routine production of gallium-68 labeled peptides. Appl Radiat Isot.

[15] Maina T (2016). Preclinical and first clinical experience with the gastrin-releasing peptide receptor-antagonist [68Ga]SB3 and PET/CT. Eur J Nucl.

[16] Baum RP (2015). First-in-human study demonstrating tumor-angiogenesis by PET/CT imaging with Ga-68-NODAGA-THERANOST, a high-affinity peptidomimetic for alpha(v)beta(3) integrin receptor targeting. Cancer Biother Radiopharm.

[17] Miller SA, Dykes DD, Polesky HF (1988). A simple salting out procedure for extracting DNA from human nucleated cells. Nucleic Acids Res.

[18] Mao YJ, Sheng XR, Pan XM (2007). The effects of NaCl concentration and pH on the stability of hyperthermophilic protein Ssh10b. BMC Biochem.

[19] Dominy BN, Perl D, Schmid FX, Brooks CL (2002). The effects of ionic strength on protein stability: the cold shock protein family. J Mol Biol.

[20] Lindman S (2006). Salting the charged surface: pH and salt dependence of protein G B1 stability. Biophys J.

[21] Šimeček J, Hermann P, Wester HJ, Notni J (2013). How is ^68^Ga labeling of macrocyclic chelators influenced by metal ion contaminants in ^68^Ge/^68^Ga generator eluates?. ChemMedChem.

[22] Chakravarty R, Chakraborty S, Dash A, Pillai MR (2013). Detailed evaluation on the effect of metal ion impurities on complexation of generator eluted ^68^Ga with different bifunctional chelators. Nucl Med Biol.

[23] (2010). 2.4.8 Heavy Metals. European Pharmacopoeia.

